# MRI-compatible electromagnetic servomotor for image-guided medical robotics

**DOI:** 10.1038/s44172-022-00001-y

**Published:** 2022-05-26

**Authors:** Lorne W. Hofstetter, J. Rock Hadley, Robb Merrill, Huy Pham, Gabriel C. Fine, Dennis L. Parker

**Affiliations:** Department of Radiology and Imaging Sciences, University of Utah School of Medicine, 30 North 1900 East #1A071, Salt Lake City, UT 84132 USA

**Keywords:** Magnetic resonance imaging, Biomedical engineering, Electrical and electronic engineering, Mechanical engineering

## Abstract

The soft-tissue imaging capabilities of magnetic resonance imaging (MRI) combined with high precision robotics has the potential to improve the precision and safety of a wide range of image-guided medical procedures. However, functional MRI-compatible robotics have not yet been realized in part because conventional electromagnetic servomotors can become dangerous projectiles near the strong magnetic field of an MRI scanner. Here we report an electromagnetic servomotor constructed from non-magnetic components, where high-torque and controlled rotary actuation is produced via interaction between electrical current in the servomotor armature and the magnetic field generated by the superconducting magnet of the MRI scanner itself. Using this servomotor design, we then build and test an MRI-compatible robot which can achieve the linear forces required to insert a large-diameter biopsy instrument in tissue during simultaneous MRI. Our electromagnetic servomotor can be safely operated (while imaging) in the patient area of a 3 Tesla clinical MRI scanner.

## Abstract (web summary)

Conventional electromagnetic servomotors are incompatible with MRI scanners. Here, Lorne Hofstetter and colleagues design and build an electromagnetic servomotor constructed from non-magnetic components and test their device in a robot operating in a clinical MRI during imaging.

## Introduction

Magnetic resonance imaging (MRI) can volumetrically image the human body in a non-invasive manner without the use of ionizing radiation^1^. The ability to visualize anatomical structure and pathology of soft tissues in exquisite detail, as well as provide functional information, has made MRI indispensable for the preoperative planning of neurosurgeries^2–5^, orthopedic procedures^6,7^, tissue biopsies^8–10^, and cancer therapies^11–14^. However, preoperatively acquired images can quickly become useless due to procedure-induced changes in the tissue geometry or environment. The introduction of needles, resection of tissues, or performance of a craniotomy to gain surgical access to the brain can result in shifting and deformation of soft tissues in the area of interest^15–19^. This tissue shift is particularly problematic for procedures where targeting accuracy is paramount to achieving a favorable outcome^5,20^ or when intraprocedural discrimination between diseased and healthy tissue relies on advanced imaging techniques such as MRI^14,18,21^.

The development of intraoperative MRI emerged to address limitations associated with using static preoperative imaging for surgical guidance. In 1994, an open 0.5 Tesla (T) MRI design was introduced that allowed direct surgical access to the patient during imaging^22,23^. The benefits of this surgical approach quickly became apparent in the resection of glioma brain tumors where maximally resecting the tumor while preserving eloquent brain regions was shown to improve survival^18,24,25^. More recently, the improved image resolution and widespread availability of closed-bore and high-field scanners (1.5 T and 3 T) has driven their use for intraoperative MRI^21^. However, the closed-bore nature of these systems (60–70 cm bore diameter) limits surgical access to the patient during imaging. Freehand approaches are possible but are ergonomically difficult and can require the physician to reach up to 1 meter into the scanner bore for access. As a work around, patient transport to the imaging department with the MRI or operating rooms equipped with a mobile MRI system^26^ are used intraoperatively to confirm critical steps during a variety of procedures. However, this paradigm of move-to-image is reactionary and does not enable concurrent intraoperative imaging for real-time guidance.

To compensate for limited patient access in closed-bore MRI scanners, medical robotic systems have been developed that can operate safely in the scanner bore^16,27–33^. The aim of such systems is to combine the precision of robotic-assisted procedures with the clinical benefit of high-resolution intraoperative MRI. However, design of these medical systems is complicated by the strong magnetic field generated by the superconducting magnet of the MRI system. Traditional electromagnetic servomotor actuators that have been refined and vetted over decades of use in industrial automation and commercial medical robots are inherently incompatible with MRI. Ferromagnetic and magnetic material used by conventional electromagnetic actuators can become dangerous projectiles if brought near the magnetic field of the MRI scanner. Hence, to date, medical robots that can operate in the MRI have relied on non-magnetic pneumatic and piezoelectric actuator technologies^34,35^. However, the limited accuracy of non-magnetic pneumatic actuator technologies that utilize long transmission lines and the potential for oscillation and overshoot^27,36^ make their use unsuitable where high precision is paramount. The electromagnetic noise generated by the operation of commercially available piezoelectric actuator technologies can interfere with the sensitive receiver hardware of the MRI. These actuators when operating simultaneous with MRI have been shown to reduce the image signal to noise ratio (SNR) by 26–80%^37–39^. While specially designed controllers have been used to keep this SNR degradation to below 15%^27^, achieving dynamic and smooth proportional actuation via closed-loop control of piezoelectric actuators is not trivial. The inability to use the electromagnetic actuation principles that are mainstays of industrial automation has limited the development, functionality, and adoption of medical systems that combine the benefits of robotic precision with the capabilities enabled by high-resolution intraoperative MRI.

Although the magnetic fields of the MRI scanner can hinder the use of conventional electromagnetic actuators, these strong fields can be leveraged to enable novel actuation strategies. The time-varying magnetic fields produced by the MRI gradient coils have been used to propel a ferromagnetic core through fluids and arteries with possible applications for microdevice delivery within the cardiovascular system^40,41^. Pulsed magnetic field gradients from the gradient coils of an MRI system were used to navigate iron oxide labeled macrophages in a living organism to deliver targeted therapies^42^. MRI gradient coils have also been used to drive rotary motion in an untethered motor with an iron-core rotor^43^. The fringe-field of the MRI scanner was used to navigate a lead wire inside the cardiovascular system by controlling the position and orientation of the animal relative to the fringe-field^44^. These efforts illustrate several methods by which components of the MRI system can be leveraged for actuation. However, a hybrid actuator system that enables traditional rotary servomotor functionality to be operated during imaging without the need to modify or design special imaging sequences and protocols has not been achieved.

In this study we present a servomotor that is constructed from non-magnetic materials and is able to unlock the paradigm of utilizing electromagnetic servomotors in close proximity to the magnetic field of the MRI system during imaging. Actuation torque is produced by harnessing the interaction between electrical currents in the servomotor armature and the magnetic field generated by the superconducting magnet of the MRI scanner. We show that this actuator design can be operated simultaneously with MRI without degrading image quality and that an optical rotary encoder and servomotor controller enable closed-loop control. We then demonstrate, in a proof-of-concept MRI-compatible surgical robot, that this servomotor can be used to drive a biopsy introducer to a target of interest while imaging at 5 frames per second.

These results constitute an important step towards highly functional robotic systems that can be used to perform interventional procedures under concurrent intraoperative MRI guidance.

## Results

### MRI-compatible direct current motor concept

**Fig. 1:**
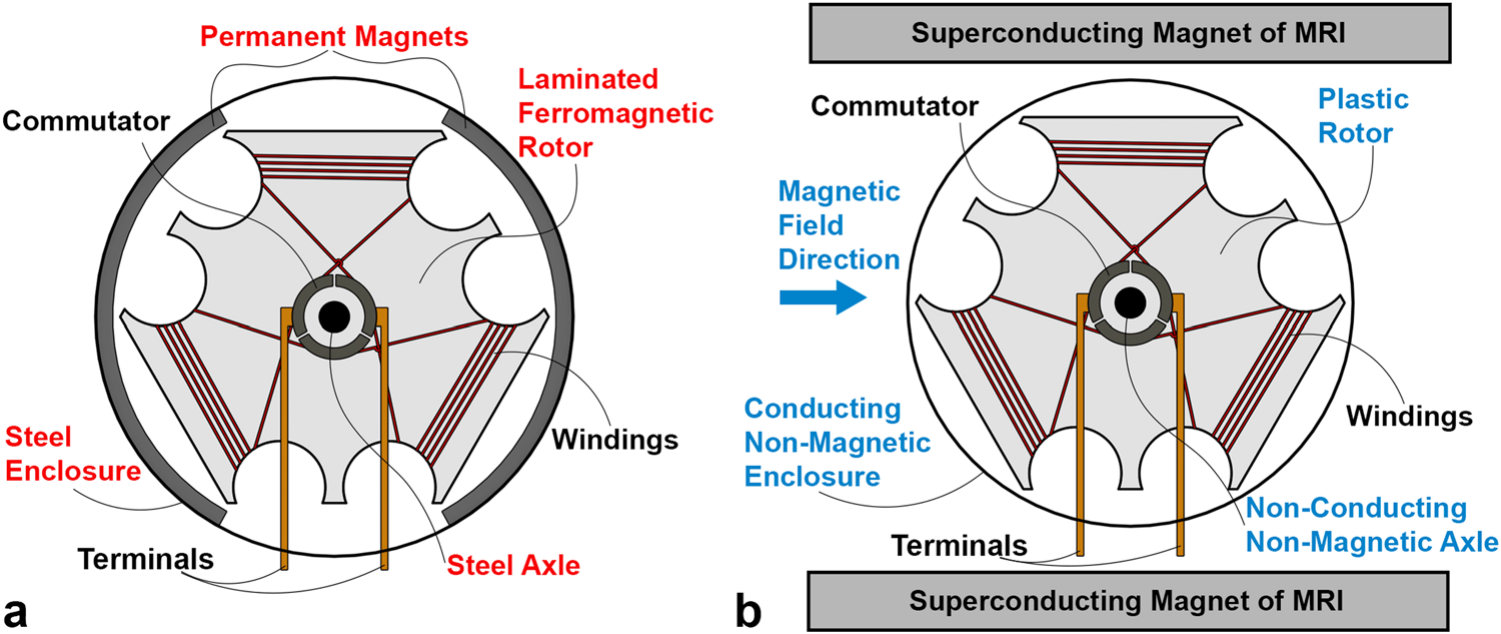
Conventional and MRI-compatible electromagnetic direct current motors. **a** Schematic of conventional direct current (DC) motor. Red labels denote components that are inherently incompatible near strong magnetic fields. **b** Schematic of MRI-compatible DC motor concept. Static field of main superconducting magnet is used instead of permanent magnets for torque generation. All magnetic and ferromagnetic materials are replaced by non-magnetic counterparts (blue text).

The conventional direct current (DC) commutator motor (Fig. 1a) is comprised of magnetic and ferromagnetic materials which can become hazardous projectiles if brought near the superconducting magnet of an MRI scanner. While many of the magnetic components can be replaced by non-magnetic counterparts, two serve important electromagnetic functions. Permanent magnets inside the motor housing (Fig. 1a) produce a static magnetic field that interacts with the electrical current in the rotor windings to generate rotary actuation. The rotor (Fig. 1a) is made from ferromagnetic laminations which focus the magnetic flux and thereby enhances the torque generation between rotor windings and the permanent magnets. While these two magnetic motor components serve both necessary and useful functions, their use near the patient area of MRI systems is inherently incompatible due to the strong forces exerted on the components by the field of the MRI system.

Figure 1b presents an electromagnetic motor concept that can operate within the bore of MRI systems and does not require the use of permanent magnets or a ferromagnetic rotor. The motor is designed to utilize the magnetic field generated by the superconducting magnet of the MRI scanner, which obviates the need for permanent magnets. Since the strong magnetic field (1.5 T–3 T for standard clinical systems) is homogeneous within the bore and extends well beyond the patient area, the use of a ferromagnetic rotor to focus magnetic flux is not needed to maintain a strong magnetic flux density at the rotor windings. Removing the ferromagnetic rotor has additional benefits in that it eliminates unwanted motor cogging torque that is associated with the reluctance of ferromagnetic materials^45^. Reduction in motor cogging can be particularly important for robotic applications requiring high precision.

**Fig. 2:**
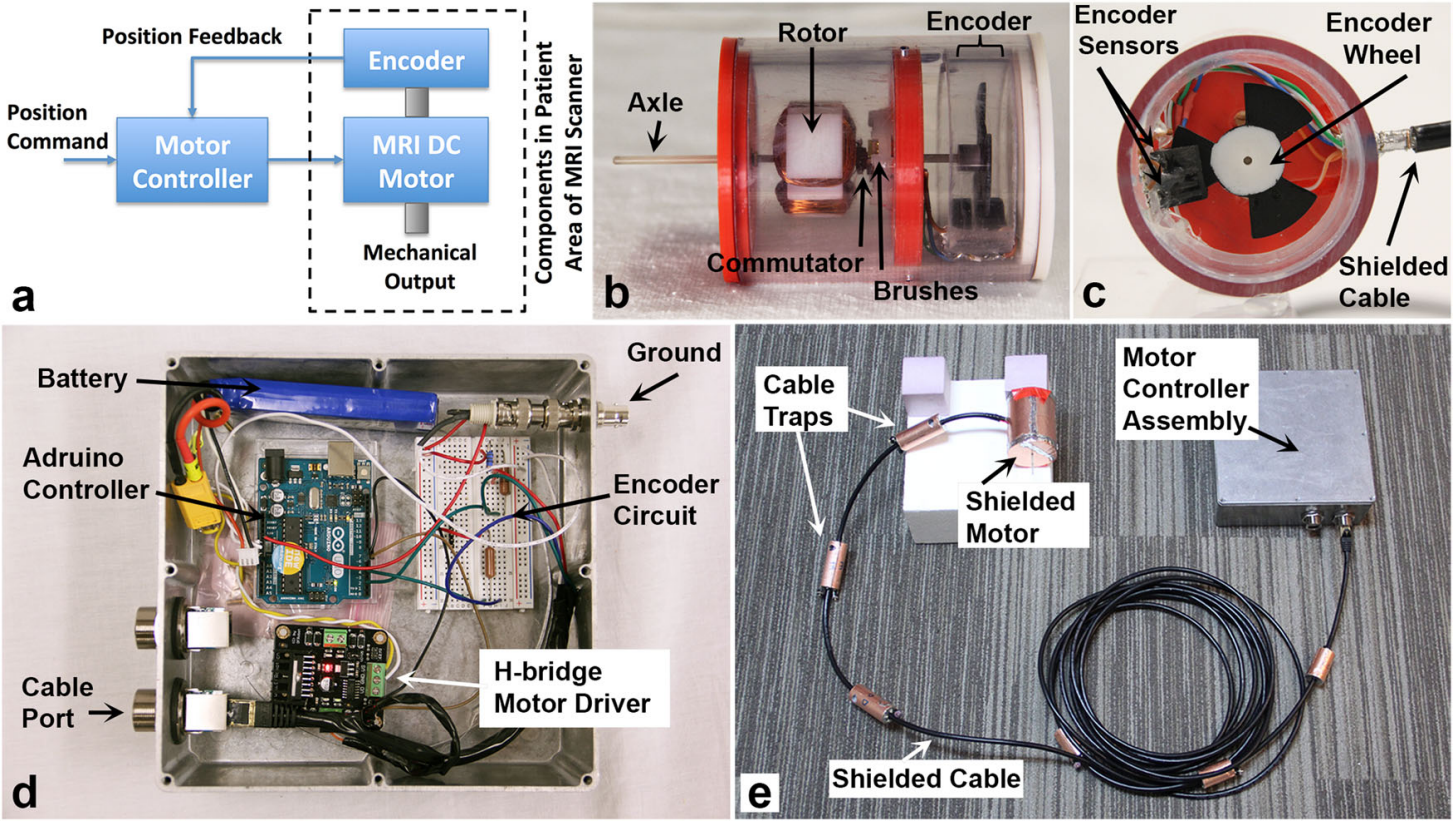
MRI-compatible servomotor. **a** Block diagram of the MRI-compatible electromagnetic servomotor concept that enables closed-loop rotary actuation. **b** Photograph of servomotor without EMI shielding. Servomotor includes the MRI-compatible DC motor described in Fig. 1b and an MRI-compatible encoder. **c** End view of the servomotor showing the two TOSwPO encoder sensor units and an encoder disk divided into 60° increments. **d** Photograph of motor controller assembly and associated sub-components. **e** Photograph of servomotor unit connected to motor controller assembly. All EMI shielding including faraday cage around servomotor and cable traps are used in this final configuration.

The torque on a single rotor winding of the motor concept in Fig. 2b is given by $\vec{T}=\vec{M}\times \vec{B}$ where $\vec{M}$ is the magnetic dipole moment generated by the winding and $\vec{B}$ is the magnetic field produced by the MRI scanner. The dipole moment generated by the winding loop is $\vec{M}={nI}\vec{A}$ where $n$ is the number of turns in the winding, $I$ is the electrical current, and $\vec{A}$ is a vector whose magnitude is the cross-sectional area of the winding and direction is normal to the winding plane. The torque component directed along the motor shaft produces rotary actuation about the axle. The magnitude of the torque about the axle is given by

1 $${T}_{s}=\hat{s}\cdot \left(\vec{M}\times \vec{B}\right)$$

where $\hat{s}$ is a unit vector along the direction of the motor shaft and $\cdot$ denotes the dot product.

Inside an MRI scanner, the magnetic field term $(\vec{B})$ in Eq. (1) is dominated by the static ${B}_{0}$ field which is at least an order of magnitude larger than the field produced by the imaging gradient coils. If we select our coordinate system so that the static ${B}_{0}$ field is oriented along the z direction and neglect the smaller field contributions from the imaging gradient coils, Eq. (1) simplifies to

2 $${T}_{s}=\left({M}_{y}\,{{\cos }}\,\phi -{M}_{x}\,{{\sin }}\,\phi \right){B}_{0}\,{{\sin }}\,\theta$$

where $\phi$ and $\theta$ are the azimuthal and polar angles specifying the orientation of the motor axle in spherical coordinates, ${B}_{0}$ is the magnitude of the static magnetic field, and ${M}_{x}$ and

${M}_{y}$ are the *x* and *y* components of the dipole moment generated by the current in the rotor loop winding.

Equation (2) shows that the motor shaft torque not only depends on the orientation and current in the rotor loop (which determines the magnitude of ${M}_{x}$ and ${M}_{y}$) but also on the orientation of the motor shaft. This positional dependence is important to consider when designing multi-degree of freedom systems that may require the orientation of the servomotor axle to change during operation. Let us consider the case shown in Fig. 2b where each coil loop on the rotor is perpendicular to the motor axle. For $\theta =0^\circ$, the motor axle is parallel to the ${B}_{0}$ field and zero shaft torque is generated in this orientation. The orientation that generates the maximum shaft torque is achieved when the axle is perpendicular to the ${B}_{0}$ field direction (i.e., $\theta =90^\circ$). Since the rotor loops are orthogonal to the axle direction, it can be shown from Eq. (2) that the motor torque is unaffected by the choice of azimuthal angle if $\theta =90^\circ .$

### MRI-compatible electromagnetic servomotor design and performance

We combined the electromagnetic motor concept in Fig. 1b with a non-magnetic optical encoder and motor controller (schematic in Fig. 2a) to achieve closed loop servomotor functionality in the MRI. Supplementary Fig. 1 shows the servomotor components prior to assembly and the assembled servomotor is shown in Fig. 2b with an end view of the servomotor encoder shown in Fig. 2c. The encoder consists of two transmissive optical sensor with phototransistor output (TOSwPO) units that detect changes in position and direction of the 3-leaf encoder disk attached to the motor axle. The encoder subdivides each axle revolution into 12 increments. The encoder circuit used to detect changes in the TOSwPO sensors is in the upper right of Fig. 2d and the corresponding circuit diagram is shown in Supplementary Fig. 2. Control of rotor position is achieved using a proportional integral (PI) controller implemented on the Arduino microcontroller (shown in Fig. 2d) which receives inputs from the encoder circuit. Speed and directional control of the motor is achieved using an H-bridge controller (shown in Fig. 2d) which sends a pulse-width modulation (PWM) control signal to the servomotor. A shielded Cat7 ethernet cable electrically connects the servomotor to the controller unit. The final motor control and servomotor assembly including all electromagnetic interference (EMI) shielding and radio frequency (RF) cable traps is shown in Fig. 2e.

Connecting a 7.4 Volt (V) lithium polymer (LiPo) battery (Hobbyking, Hong Kong) to the DC motor terminals induces rotary motion of the motor rotor and axle (Supplementary Movie 1). Supplementary Movie 2 shows the motor operating at a different azimuthal orientation angle in the MRI bore for $\theta =90^\circ$. Control of the servomotor using closed-loop-feedback control is demonstrated in Supplementary Movie 3. After a command was issued by the motor controller to increment by 120 steps, return to the desired setpoint was maintained even when the motor shaft was forcefully perturbed away from the setpoint. These results demonstrate closed-loop control of an electromagnetic servomotor constructed from non-magnetic components and operated inside the MRI scanner bore.

Servomotor stall torque (measured using apparatus shown in Supplementary Fig. 3) and unloaded shaft speed when powered by the 7.4 V battery and operated at field strength of 2.89 T are shown in Table 1. Servomotor diameter and length are 58, and 74 mm, respectively.

**Table. 1:** Servomotor performance.

	No Load	Stall
Current (A)	0.07	1.58
Voltage (V)	8.29	7.65
Speed (rpm)	1524	–
Torque (mNm)	–	73.1

Measurements at MRI scanner isocenter where servomotor axle is perpendicular to direction of the scanner magnetic field ($\theta =90^\circ$).

**Fig. 3:**
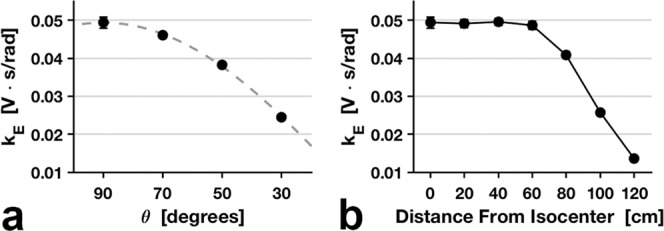
Back EMF constant depends on servomotor axle orientation and distance from MRI scanner isocenter. The servomotor back EMF constant (${k}_{{{\mbox{E}}}}$) with respect to the servomotor axle orientation $(\theta)$ is shown in (**a**). All measurements in (**a**) were performed with the servomotor located at the MRI scanner isocenter. Dotted line in (**a**) is the value of ${k}_{{{\mbox{E}}}}$ (measured at $\theta =90^\circ$) multiplied by ${{\sin }}(\theta )$. Agreement between measurement data (solid circles) and dotted line confirms the angular dependence predicted by Eq. (2) The back EMF constant with respect to the distance between the servomotor location and the MRI scanner isocenter is shown in (**b**). All measurements in (**b**) were performed for $\theta =90^\circ$. Error bars in (**a**) and (**b**) denote the standard deviation of 9 measurements obtained for each scenario.

The servomotor performance was further characterized by measuring the back electromotive force (EMF) constant, ${k}_{{{\mbox{E}}}}$, for different servomotor axle orientations and servomotor distances from the MRI scanner isocenter. In a DC motor, ${k}_{{{\mbox{E}}}}$ is equivalent to the torque constant, ${k}_{{{\mbox{T}}}}$, which describes the linear relationship between the supplied rotor winding current and the axle torque produced^46^. Figure 3a shows that ${k}_{{{\mbox{E}}}}$ is maximum when the motor axle is perpendicular (i.e., $\theta ={90}^\circ$) to the ${B}_{0}$ field of the scanner. The measured ${k}_{{{\mbox{E}}}}$ decreased for the three other orientation angles and this decrease followed a ${{\sin }}(\theta )$ dependence as predicted by Eq. (2). Figure 3b shows that for $\theta ={90}^\circ$, ${k}_{{{\mbox{E}}}}$ remains constant when the motor is located within 60 cm of the MRI scanner isocenter. At 1 meter from isocenter, ${k}_{{{\mbox{E}}}}$ dropped to 52% of the value at isocenter. It is important to highlight that the ${k}_{{{\mbox{E}}}}$ with-respect-to distance from isocenter curve will be unique for different MRI scanner designs and manufacturers. However, since all systems strive to retain a constant and highly uniform magnetic field near the imaging region, it is reasonable to assume a constant ${k}_{{{\mbox{E}}}}$ and a predictable current-torque response if the servomotors are operated in close proximity to the imaging region.

### Simultaneous imaging and servomotor operation

The MRI transmit/receive hardware is extremely sensitive to radio frequency (RF) energy. Sources of electromagnetic noise near the proton Larmor frequency (123.23 MHz@2.89 T) can substantially degrade imaging performance by introducing unwanted electromagnetic signal into the image receiver hardware. The making and breaking of electrical contacts between the brushes and commutator during servomotor operation generates broadband radio frequency (RF) noise over a wide frequency band and this noise source can degrade MRI image quality if not sufficiently mitigated (Supplementary Fig. 4). The H-bridge motor controller uses pulse width modulation to control the effective voltage signal to the motor leads. The square voltage waveforms associated with the PWM control scheme can generate broadband RF noise which is a potential noise source that can contribute to image degradation.

To minimize interactions between the servomotor and the MRI system, three critical design aspects were incorporated into the servomotor and controller shown in Fig. 2e. First, EMI shielding principles were used to prevent broadband energy produced by the H-bridge controller and motor brushes from radiating to the MRI receiver hardware. The servomotor was housed in a continuous copper shield (Fig. 2e). A 2 mm hole in one end of the shield allowed the motor axle to penetrate the housing. The motor controller unit and associated electronics were enclosed in a grounded and shielded box. Power and control signals between motor and motor controller unit were transmitted by a double-shielded Cat7 ethernet cable (4 twisted pairs, one pair supplies current to run the motor, one pair is used to power the encoder diodes, and two pairs are used to return sensor signals to the motor controller). The shield of the Cat7 cable was soldered to the motor shield and electrically connected to the grounded shielded box of the motor controller using RJ45 connectors (Fig. 2e). Second, to prevent the motor axle from acting as an antenna and radiating noise contained inside the motor faraday cage, a low conductivity 2 mm diameter composite axle was used. Third, six cable traps tuned to the proton Larmor frequency were installed and spaced 15 cm apart on both ends of the shielded cable to prevent any RF energy near 123.23 MHz from traveling on the cable shield. These cable traps serve two important functions: (1) to prevent unwanted common mode currents at the Larmor frequency from introducing unwanted electrical noise into the imaging region of the MRI system and (2) to minimize potential heating of the shielded cable by the MRI transmit field.

**Fig. 4:**
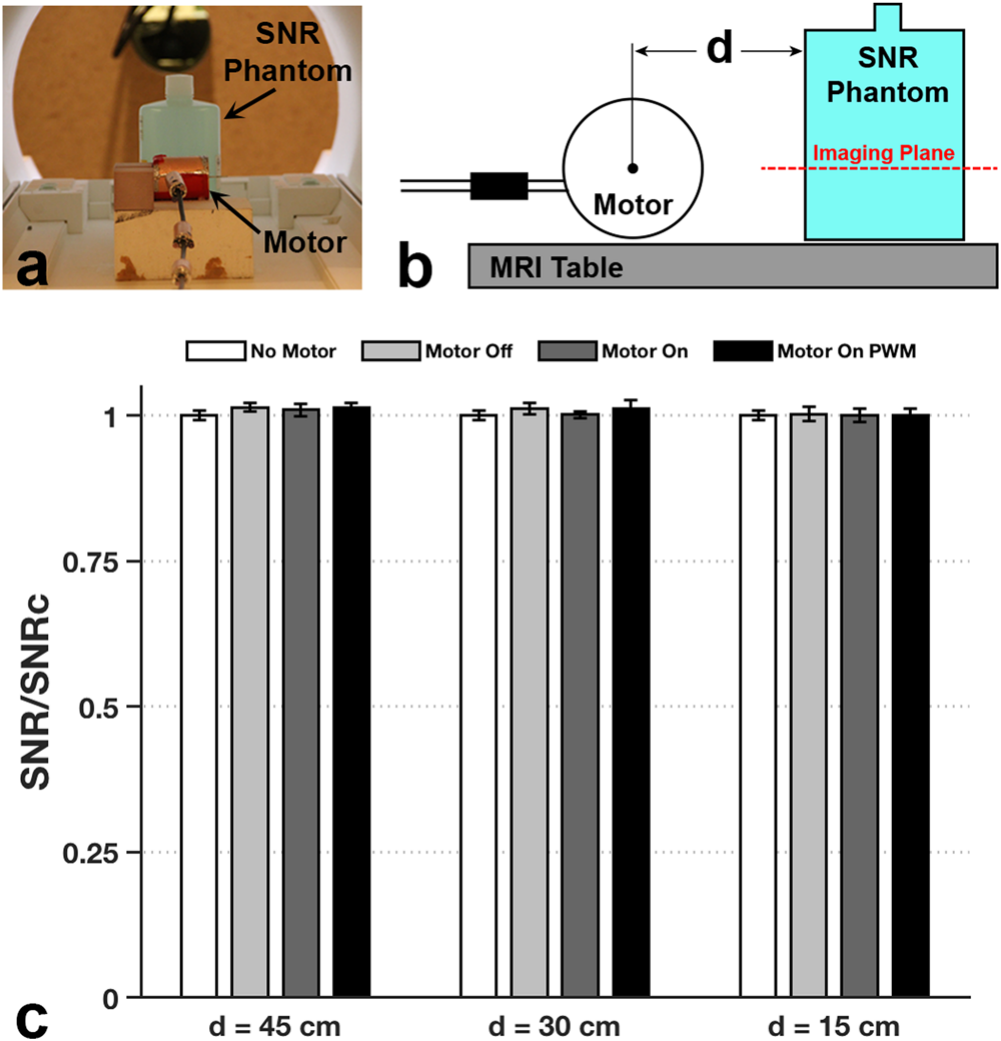
Imaging largely unaffected by servomotor presence and operation. **a** Photograph depicting orientation of servomotor and phantom for SNR imaging tests. **b** Side view schematic of (**a**) where d denotes the distance between the phantom and servomotor axle. **c** Normalized SNR image quality measurement for d = 45, 30, and 15 cm. For each distance three scenarios were tested: motor off, motor on (driven by DC power supply), and motor on (driven by unfiltered PWM signal from H-bridge controller where a 50% square wave duty cycle was used). Error bars denote standard deviation of 12 SNR measurements obtained for each scenario. For all tested scenarios, SNR differed from the control measurement (no motor) by less than 1.5%.

Results from Fig. 4 demonstrate that use of the EMI design strategies listed above limits unwanted interactions between the MRI system and the operating servomotor. Measured signal to noise ratio (SNR) of images acquired using MRI differed from control by no more than 1.5% for a range of test configurations during the servomotor operation. The motor position was varied between 45 and 15 cm and the motor was powered by both an H-bridge motor controller and a DC voltage supply. Figure 4c shows that for all test conditions and distances, measured image SNR was remarkably similar to imaging in the absence of the servomotor unit. Thus, the ability to simultaneously image with MRI and operate electromagnetic servomotor actuators using conventional actuation principles and motor controllers was demonstrated.

### Proof of concept biopsy introducer robot

The accuracy of using focal biopsy techniques to classify cancer risk critically depends on the lesion targeting accuracy of the procedure^47^. Current MRI-guided prostate and breast biopsy procedures employ the use of a grid template and pre-treatment images to guide placement of an introducer sheath in order to obtain access to the desired biopsy target. This sheath placement is an iterative process that consists of moving the patient in and out of the scanner bore for imaging and then manually adjusting the introducer sheath position until the desired target is reached. Once the target is reached, the biopsy can subsequently be obtained.

**Fig. 5:**
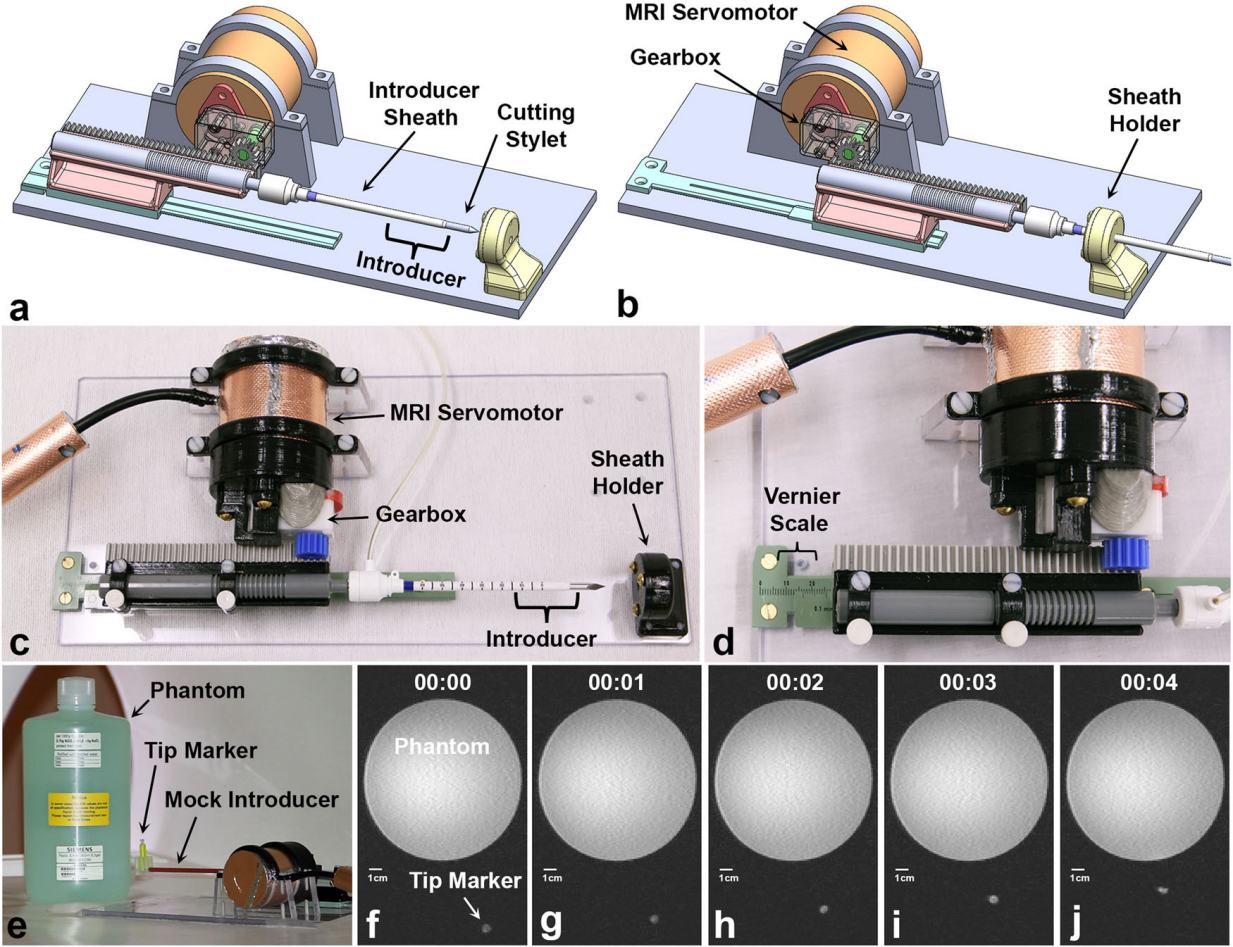
Biopsy introducer robot actuated by MRI-compatible electromagnetic servomotor. **a** Schematic of single degree-of-freedom biopsy introducer robot prior to introducer insertion. **b** Schematic showing maximum insertion depth. Sheath holder allows introducer sheath position to be maintained during removal of cutting stylet. **c** Photograph of biopsy introducer robot with MRI-compatible servomotor. **d** Zoomed in photograph showing Vernier scale on a linear slide with 0.1 mm increments. This Vernier scale is used for system calibration when the robot is first powered on. **e** Photograph of biopsy robot in the MRI scanner where introducer was replaced with a mock introducer and a tip marker that can be imaged with MRI. Servomotor was used to advance the mock introducer in a controlled manner during continuous imaging at 5 frames per second. Simultaneously acquired coronal images showing movement of tip marker are shown at 1 s increments during actuation in (**f**–**j**).

To demonstrate the ability of our MRI-compatible electromagnetic actuator to control a surgical tool during imaging, a proof-of-concept surgical robot was constructed that can place a 9-gauge biopsy introducer sheath under real-time MRI-guidance. An illustration of the 1-degree-of-freedom robot is shown in Fig. 5a, b where Fig. 5a shows the introducer in a retracted position, and Fig. 5b shows the introducer in the fully inserted position. The constructed robot is shown in Fig. 5c. The Vernier scale on the introducer stage (shown in Fig. 5d) is used to calibrate the position of the linear stage controlling the introducer placement. Gearing connecting the servomotor output to the linear stage results in a maximum linear stage speed of 10 mm/s and a maximum insertion force of 585 N (131 lbs). The maximum range of the linear stage travel is 10 cm.

Simultaneous actuation of the linear stage and imaging with MRI was demonstrated using the setup in Fig. 5e. The introducer shown in Fig. 5c was replaced by a mock introducer (in Fig. 5e) which has an imaging fiducial marker at the position of the mock needle tip. Using a 5 frame/second imaging protocol, imaging was performed during movement of the mock introducer. Images separated by 1 s are shown in Fig. 5f–j, which demonstrates that high-frame-rate imaging can be used to visualize robot actuation. This simultaneous imaging and actuation are shown in Supplementary Movie 4. Supplementary Movie 5 shows that the orientation of the robot relative to the magnetic field of the MRI can be modified to achieve movement of the introducer along different insertion orientations.

**Fig. 6:**
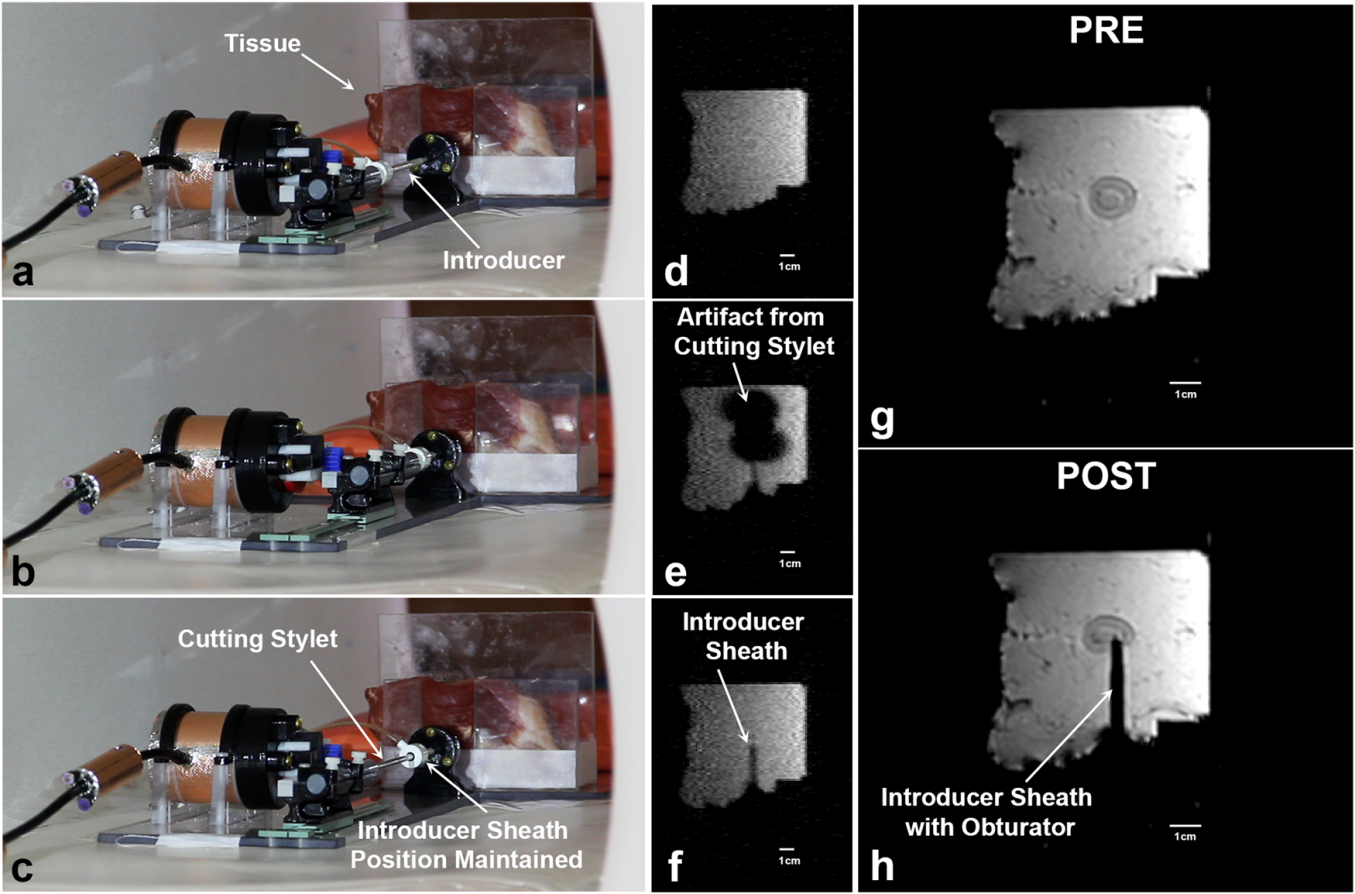
Biopsy introducer robot with MRI-compatible servomotor places introducer sheath at desired target location in ex vivo tissue. Photographs of introducer robot prior to introducer insertion (**a**), at target insertion depth (**b**), and during cutting stylet removal (**c**) are shown. The corresponding simultaneous MR images obtained during robot operation are shown in (**d**), (**e**), and (**f**), respectively. **g** Sagittal MR image of tissue sample showing target lesion prior to biopsy introducer placement. **h** Sagittal MR image following introducer sheath placement showing that desired placement position of introducer sheath was achieved.

The MRI-compatible biopsy insertion robot was then used to place a 9-gauge introducer sheath to a predetermined tissue target during continuous imaging. Volumetric MRI was performed prior to needle insertion to determine the desired introducer sheath placement location in imaging coordinates. The robot was commanded under one continuous operation to drive the cutting stylet and introducer sheath from the initial position (Fig. 6a) to the desired target (Fig. 6b) and then to remove the cutting stylet from the introducer sheath (Fig. 6c). The corresponding real-time images for each of these steps are shown in Fig. 6d–f with the pre and post introducer sheath placement images shown in Fig. 6g, h, respectively. This ex vivo tissue experiment demonstrates that a proof-of-concept surgical robot powered by the MRI-compatible electromagnetic servomotor can drive a large diameter introducer through tissue to reach a desired and predetermined target. The full sequence for the introducer sheath placement with simultaneous MRI is shown in Supplementary Movie 6.

## Discussion

We have presented an electromagnetic servomotor that can safely operate in the patient area of MRI scanners. The servomotor was constructed from non-magnetic materials and hence is not a potential projectile hazard in this environment. Rotary actuation was generated by leveraging the interaction between electrical currents in the servomotor rotor windings and the superconducting magnetic field of the MRI scanner. Closed-loop position control of the servomotor axle was achieved using an optical encoding method. The servomotor was specifically designed to minimize EMI so that simultaneous robotic actuation and imaging with MRI could be performed. Utilizing this servomotor, a proof-of-concept robot was constructed and tested to show that the linear forces required to insert a large-diameter (9-gague) biopsy introducer in an ex vivo tissue sample could be achieved during simultaneous serial imaging with MRI.

A key benefit of this MRI-compatible actuator technology is that it uses standard electromagnetic actuation principles and control hardware commonly used in commercial medical robots and industrial automation to produce high-torque rotary actuation. The ability to draw on this prior body of work with respect to electromagnetic motor systems will simplify the future development of highly functional MRI-compatible robotic systems.

As a second major benefit, the servomotor enables simultaneous imaging and robotic actuation. Under the current paradigm of high-field intraoperative MRI, either the patient or MRI scanner is moved into position for imaging to confirm critical steps in the procedure. This repositioning is both time consuming and reactionary in that it does not enable real-time decision making in an ever-changing surgical environment. For example, brain shift during neurosurgery occurs continuously and unpredictably throughout the procedure^48,49^, and for procedures that can benefit from the visualization capabilities of intraoperative MRI, such as glioma tumor resections and needle-based procedures, robotic systems that enable serial and concurrent imaging could improve procedural precision and safety. MRI-guided prostate biopsy studies have shown that needle bending and skin-needle interactions during manual insertion of the biopsy needle can result in targeting errors that are on the order of half a centimeter^17,50^. A robotic-assisted needle insertion approach that utilizes feedback from concurrent intraoperative MRI could be used to visualize and correct for needle bending and tissue deformation in real-time during needle insertion. This would minimize the need for needle reinsertion following a failed targeting attempt. The MRI-compatible servomotor presented in this work may enable the use of 4D intraoperative MRI where volumetric MRI is performed serially in time and is used to inform control of robotic systems during medical procedures.

Leveraging the strong magnetic field of the MRI system to actuate servomotors enables safe electromagnetic actuation in the patient area of the scanner. The 1.5 or 3 Tesla magnetic flux density produced by conventional closed-bore MRI systems unlocks the possibility of small footprint servomotor actuators. Current state of the art electromagnetic motors use rare-earth metals such as Neodymium to generate the magnetic field that interacts with electrical currents in the motor windings. The remanence of a Neodymium magnet is less than 1.3 Tesla^51^ and the resulting magnetic flux density in air rapidly decreases with distance from the magnet surface. In a servomotor design that leverages the superconducting field of the MRI, the in-air magnetic flux density that is produced is much larger than the in-air magnetic field that can be generated by the rare-earth permanent magnets. Hence, an MRI-compatible servomotor as described can in principle be constructed with a much smaller physical footprint while maintaining the same peak operating performance of state-of-the-art permanent magnet servomotors. This potential for miniaturization is important for developing robotics system that can operate in the limited space of closed-bore MRI systems.

Using the strong magnetic field of the MRI for actuation does place some constraints on the orientation of the servomotor during operation. The MRI-compatible servomotor presented here used a mechanical commutation scheme which requires that the rotational alignment of the motor brushes and static magnetic field of the MRI be maintained for optimal motor performance. However, for applications where the rotational alignment of the servomotor housing about the axle axis of the servomotor cannot be maintained, a redesign of the commutator would allow consistent operation for any rotational alignment about the axle axis. Instead of mechanical commutation, the use of slip rings to provide continuous electrical connections to the rotor, combined with the electrical commutation control schemes widely used by brushless permanent magnet motors would eliminate the need to maintain a strict commutator orientation.

Other translations of the servomotor that change the amount of flux passing through the rotor loop windings will alter the achievable torque and speed of the motor. If the servomotor coils are powered by a constant voltage source, a change in the motor orientation that decreases the magnetic flux through the rotor loops will decrease the maximum stall torque and increase the unloaded motor speed. This effect is characterized by the back EMF constant (${k}_{{{\mbox{E}}}}$) measurements in Fig. 3. The value of ${k}_{{{\mbox{E}}}}$ depends on ${{\sin }}(\theta )$ where $\theta$ is the polar angle describing the servomotor axle position relative to the ${B}_{0}$ field direction. When the axle is perpendicular to the ${B}_{0}$ field ($\theta =90^\circ$) the magnetic flux passing through the rotor loop windings is maximum and the maximum ${k}_{{{\mbox{E}}}}$ is achieved. Figure 3b shows that for large distances from isocenter (greater than 60 cm), ${k}_{{{\mbox{E}}}}$ decreases. This decrease is due to the fact that the magnetic field strength starts to decrease substantially outside the bore of the MRI scanner. The variation in ${k}_{{{\mbox{E}}}}$ with position and polar angle can affect the motor torque and control characteristics. However, if the servomotor is sufficiently sized to accommodate the different orientations needed for a robotic application, this change in operating conditions with orientation can be readily controlled by the closed-loop nature of the servomotor controller. This is demonstrated by Supplementary Movies 4 and 5 where orientation of the proof-of-concept robot was changed with respect to the superconducting field of the MRI system. Supplementary Movies 1 and 2 demonstrate that changes in servomotor orientation that do not change the rotational alignment of the brushes or alter the magnet flux seen by the rotor windings have no impact on the motor operation. Given this flexibility and the possibility of using electrical commutation schemes that enable an arbitrary rotational alignment of the servomotor, a very wide range of actuation options at different orientations are possible. This flexibility could be used to enable the actuation of multi-degree of freedom robotic systems.

We also anticipate that this MRI-compatible servomotor will have important non-surgical applications. There are, for example, many non-surgical applications that require motion in the MRI scanner. These include magnetic resonance elastography^52^, which requires a vibrating pillow next to the patient, MRI compatible ultrasound devices for imaging and therapy that could be positioned robotically, and phantom studies that require internal motions to mimic physiological motion.

## Methods

The objective of this study was to demonstrate that electromagnetic actuator principles vetted and widely used in industrial automation are not inherently incompatible with MRI systems. This study design was to: (1) build an electromagnetic servomotor that uses the field generated by the superconducting magnet of a clinical MRI system for actuation to achieve controlled rotary motion; (2) measure the servomotor performance while operating in the patient area of an MRI scanner; (3) implement EMI reduction and shielding strategies to enable simultaneous operation of servomotor and MRI; (4) quantify interactions between the operating servomotor and MRI using SNR measurements; (5) construct a proof of concept biopsy introducer surgical robot using the presented electromagnetic servomotor design and demonstrate that real-time MR imaging can be used to track robot motion; and (6) demonstrate that a biopsy introducer robot can drive and place a 9-gauge introducer sheath to a desired target location in an ex vivo tissue sample. For all experiments in this study, the servomotor and robot were operated while in the patient area of a 60 cm diameter bore, clinical 3 T Prisma Fit MRI scanner (Siemens Medical Solutions, Erlangen, Germany).

### Servomotor construction details

Components of the prototype servomotor are shown in Supplementary Fig. 1. The motor axle was constructed from a 2 mm diameter G-10/FR4 non-conducting rod (McMaster-Carr, #8669K627). Mechanical commutator and brushes were obtained from a disassembled 280 micro 3 V–12 V DC toy motor. The support structure for the rotor windings was 3D printed from VeroWhitePlus (Stratasys, Israel). Each of three 100-turn rotor windings (~20 mm^2^ cross-sectional area) was hand wound from 30-gauge Polyamideimide magnet wire (Remington Industries, Illinois, USA). Once wound, cyanoacrylate glue was used to secure rotor windings in place. Solder was used to connect rotor windings to the commutator. The measured resistance of each rotor loop was 1.2Ω.

The outer housing of the servomotor was constructed from 57.1 mm outer diameter clear polycarbonate tubing (McMaster-Carr, #8585K28). Motor end rings were 3D printed from ABS plastic and 2 mm inner diameter Olite bushings (McMaster-Carr, #6658K411) were pressed into the motor end rings to provided support for the motor axle. Powdered graphite lubricant (Panef Corp., Milwaukee, WI) was used to minimize friction between the axle and bushings. One end ring had 3D printed details to enable proper alignment and fixation of the brushes to the end ring. A 50.8 mm outer diameter clear polycarbonate tubing (McMaster-Carr, #8585K26) that fits inside the outer housing was used to maintain alignment and proper separation of motor end rings. Two additional tight-fitting bushing (not shown in Supplementary Fig. 1) were secured to the axle to keep the rotor properly situated between the two end rings of the motor housing.

The encoder was constructed from a 3D printed ABS plastic encoder wheel (5 mm thickness, 35 mm maximum diameter). To ensure opacity of the encoder wheel, each leaf was spray coated with black paint. The two TOSwPO sensors (TCST2103, Vishay Intertechnology Inc.) were mounted to the circular support polycarbonate tubing (50.8 mm outer diameter) so that four unique states of the sensors were possible: (1) both sensors blocked by an opaque encoder leaf, (2) first sensor blocked by an opaque encoder leaf and second not blocked, (3) second blocked by an opaque encoder leaf and first not blocked,(4) neither sensor blocked. These four unique states enabled rotor motion and direction to be measured.

The constructed servomotor assembly without EMI shielding is shown in Fig. 2b, c. To install the EMI shielding, the outer polycarbonate housing of the servomotor was coated in copper shielding foil tape (3 M, #1739-17). Tape seams were soldered to ensure electrical connection between all segments. A 25-foot double-shield Cat7 ethernet cable consisting of 4-twisted pair 26-gauge wires (Tera Grand, California, USA) was used to transmit all signals between the servomotor (Fig. 2b) and motor control assembly (Fig. 2d). One twisted pair was used to supply current to the motor terminals. A second twisted pair was used to supply power to the diodes on the TOSwPO sensors. The remaining two twisted pairs communicate the TOSwPO sensor signals to the motor controller assembly. All electrical connections at the servomotor were soldered and the Cat7 cable shield was soldered to copper tape on the motor housing. An RJ45 connector on the end of the Cat7 cable distant from the servomotor enabled easy connection of wires and cable shield to the motor controller assembly.

The motor controller assembly (Fig. 1d) was housed in an 18.8 × 18.8 × 6.7 cm aluminum box. A female RJ45 connecter (PEI-genesis, PA, USA) enabled easy connection of the servomotor to the motor controller assembly. The shielded box, cable shield, and motor shield are all connected to ground via a BNC connector on the back of the box (Fig. 2d). All power to the servomotor and controller is provided by a 7.4 V 2-cell LiPo 2100 milli-amp-hour (mAh) battery (Hobbyking, Hong Kong). All control logic was implemented on the Arduino Uno Rev3. The TOSwPO sensor signals are fed as inputs to the Arduino which then sends the desired PWM control signal to a 2-amp H-bridge motor controller (DFRobot, DRI00002). Additional circuitry (see circuit diagram in Supplementary Fig. 2) used to read TOSwPO signals is located on a solderless breadboard (see Fig. 2d).

Six floating shield current suppression traps^53^ were constructed and installed 15-cm apart (Fig. 2e) on the terminal ends of the Cat7 ethernet cable in order to suppress common mode currents on the cable shield. RF traps were constructed to attenuate signals at 123.23 MHz (the proton Larmor frequency at 2.89 Tesla) to further minimize interactions between MRI transmit/receive hardware and servomotor hardware. Outer and inner diameter of traps were 22 mm and 7 mm, respectively. Two trap variants with a length of 38 mm and 57 mm were used and had a mean attenuation at 123.23 MHz of 7.4 dB and 11.3 dB, respectively.

### Encoder sensing and controller

Accurate detection of the signal from the TOSwPO sensors is required for precise servomotor control. The schematic in Supplementary Fig. 2 shows the circuit used to power the TOSwPO diodes as well as the voltage dividing sensing circuit. The voltage at the Arduino input pin for a given sensor is low when the opaque encoder wheel leaf is not blocking the transistor sensor, otherwise the voltage is high. Control software was implemented on the Arduino to continuously monitor the state of the sensor signal to allow changes in rotor position to be detected and the number of rotation increments to be counted. The Arduino was programmed to receive a command input (in number of encoder steps) from the user. A proportional integral (PI) controller was then used to generate a control signal sent to the H-bridge motor controller to achieve the desired commanded actuation.

### Servomotor performance measurements

Stall torque and unloaded motor speed were measured for the servomotor operating in the 2.89 T magnetic field of a clinical MRI scanner for the $\theta =90^\circ$ axle orientation. For both measurements, the motor was directly powered by the 7.4 V LiPo battery. A Fluke 77 and Fluke 27 multimeter were used to measure voltage across the motor leads and rotor loop current during operation. Stall torque was measured using the experimental setup shown in Supplementary Fig. 3. A mass was attached to the 2-inch diameter pulley mounted on the servomotor axle. The amount of mass was increased incrementally until the maximum lifting capacity of the motor-pulley assembly was determined. The reported stall torque is the product of the maximum lifted weight times the pulley radius. To determine the maximum motor shaft speed, the servomotor was powered in the unloaded state. The encoder hardware and associated circuitry was used to count the number of full axle revolutions occurring over a one-minute interval.

The back EMF constant, ${k}_{E}$, was calculated using the following expression from ref. ^46^

3 $$E={k}_{{{\mbox{E}}}}{\omega }_{{{\mbox{m}}}}$$

where $E$ is the EMF (in Volts) produced when turning the motor as a generator and ${\omega }_{{{\mbox{m}}}}$ is the mechanical angular speed (in radians per second) of the motor axle. To turn the servomotor as a generator inside the MRI, an MRI-safe DC motor (concept in Fig. 1b) was constructed and mechanically coupled to the servomotor shaft as is shown in Supplementary Fig. 5a. The servomotor circuitry was used to measure angular speed of the axle and a Fluke 77 multimeter to measure the EMF generated across the servomotor leads. These measurements allowed ${k}_{{{\mbox{E}}}}$ to be calculated using Eq. (3) for a variety of test conditions. The first experiment consisted of calculating ${k}_{{{\mbox{E}}}}$ of the servomotor at isocenter when the polar angle ($\theta$) of the servomotor axle orientation was varied. Supplementary Fig. 5b, c depicts how the axle alignment was determined with a protractor outside the scanner bore. Once the angular position was achieved, the MRI table was advanced until servomotor and experimental setup was at scanner isocenter. The second experiment evaluated how servomotor distance from isocenter along the z direction (for $\theta =90^\circ$) impacts ${k}_{{{\mbox{E}}}}$. For each tested condition, ${k}_{{{\mbox{E}}}}$ was measured nine times. For each of the nine measurements, the angular speed was varied between roughly 300 and 600 revolutions per minute in order to capture any axle-speed related variability in the measurement. The nine values at different angular speeds were averaged to produce the reported ${k}_{{{\mbox{E}}}}$ for each test condition.

### SNR Measurement during motor operation

Interactions between the operating servomotor and MRI were evaluated using SNR measurements. The orientation of the motor and phantom being imaged are depicted in Fig. 4b. Simultaneous imaging and motor operation was tested for three different states: (1) Motor off but located in the MRI scanner bore, (2) Motor on and powered by the 7.4 V LiPo battery, and (3) Motor on and powered by the PWM signal output from the H-bridge motor controller. The distinction between (2) and (3) was that the PWM signal from the motor controller generated a 50% duty cycle square wave voltage signal. For each test condition, image quality of the MRI was evaluated by measuring the SNR for each acquired image. SNR measurements were obtained for three different motor-phantom separation distances (d = 15, 30, 45 cm). For each separation distance, the three motor states (described above) were tested. Control SNR measurements (no motor) were also acquired with motor and motor controller completely removed from the MRI scanner room.

For SNR measurements, a 2 channel transmit/receive body coil was used to acquire a 2D gradient echo (GRE) image in the coronal plane with the following acquisition parameters: echo time/repetition time (TE/TR) = 3.58/200 ms, flip angle = 60°, field of view (FOV) = 22 cm, resolution = 1.72 × 1.72 × 5 mm, bandwidth = 260 Hz/pixel, 1 average. Images for each coil were reconstructed from raw k-space data in MATLAB (MathWorks, Natick, MA). SNR maps were formed using the noise-covariance-weighted sum of squares magnitude image reconstruction method^54–56^. Noise covariance information was calculated from pixels in the over-scan area of the image that was dominated by noise. For each scenario, 12 SNR maps were acquired. For each map, the mean SNR over the phantom cross-section was calculated and reported as a single SNR image quality metric. The mean and standard deviation of this mean SNR image quality metric was calculated and reported for each tested scenario.

### Biopsy introducer robot construction

A proof-of-concept one degree of freedom robot was constructed from non-magnetic components (Fig. 5a–d). A modified plastic Vernier caliper was used as a linear stage and the 0.1 mm scaling was used for initial calibration of the linear stage position when the robot was first powered on. The MRI-compatible electromagnetic servomotor described earlier in this paper was used for actuation. A 120:1 Plastic Gearmotor (Pololu, NV, USA) was mounted to the output axle of the servomotor using a 3D printed gearbox holder made from ABS plastic (shown in Fig. 5c). To ensure that the gearmotor was non-magnetic, the ferromagnetic steel axles in the gearbox were replaced by 2 mm diameter 316-stainless steel axles (McMaster Carr, #9298K31). A 15 tooth 15 mm diameter plastic gear (McMaster-Carr, 2262N415) was attached to the output of the gearmotor and coupled to a matching linear gear rack (McMaster-Carr, 266N57) to provide actuation of the linear stage. A 3D printed sheath holder (in Fig. 5b, c) allows the biopsy introducer sheath to be held in a fixed position during removal of the cutting stylet. To demonstrate simultaneous imaging and actuation of the robot, a mock introducer (Fig. 5e) consisting of a fiberglass rod with cylindrical fiducial marker (Hologic, Marlborough, MA) was constructed.

An ex vivo tissue experiment was performed to demonstrated accurate placement of a 9-gauge introducer (Hologic, Marlborough, MA) into a pre-specified target during simultaneous imaging with MRI. The introducer, which is comprised of a cutting stylet and introducer sheath used for MRI-guided breast biopsy procedures, was mounted onto the robot linear stage as is shown in Fig. 5c. A sheath holder (shown in Fig. 5c) that uses a rubber friction mechanism was built to both allow the insertion of the introducer and to hold the introducer sheath at the desired insertion depth during removal of the cutting stylet.

### Ex vivo experiment: tissue sample preparation and robot calibration

Ex vivo porcine loin was obtained from a local grocery store. To emulate a cancerous tissue lesion, a small incision was made and a pitted olive was embedded in the tissue. The tissue sample was placed in a tissue holder (visible in Fig. 6a) that is attached to the robot base. The robot was powered on after being placed in the MRI scanner. The Vernier scale on the linear stage was used to calibrate the initial position of the biopsy introducer when the robot was first powered on.

Next, pretreatment MRI using a 3D VIBE pulse sequence was performed with the following scan parameters: TE/TR = 2.46/7.04 ms, flip angle = 10°, field of view (FOV) = 25.6 cm, resolution = 1 × 1 × 1 mm, bandwidth = 890 Hz/pixel, 3 averages. Using the MRI console, the image coordinates of the desired biopsy target were selected from the VIBE images. This coordinate position was used by the servomotor controller to determine the number of motor increments needed to place the introducer sheath at the desired insertion depth. Following automated placement of introducer sheath and removal of cutting stylet, a plastic obturator (Hologic, Marlborough, MA) was inserted into the cutting sheath to provide improved visualization of the sheath tip position with MRI. Imaging using the same pretreatment 3D VIBE imaging protocol was performed to confirm placement location of the introducer sheath.

### Rapid MRI imaging protocols used during robot actuation

Single slice 2D MRI was used to track the mock introducer tip location during the phantom experiment and to actively monitor the introducer insertion during the ex-vivo experiment. The spine coil array mounted in the patient table was used. Pulse sequence parameters were chosen to achieve an imaging rate of 5 frames/second (0.2 s per image). For the phantom experiment, a TRUFI pulse sequence was used with the following scan parameters: TE/TR = 1.94/3.87 ms, flip angle = 45°, matrix = 256 × 62, resolution = 1.17 × 1.25 × 5 mm, bandwidth = 1149 Hz/pixel, partial Fourier in phase-encoding = 5/8, GRAPPA parallel imaging with 22 reference lines and an acceleration factor of 2. For the ex vivo experiment, a FLASH pulse sequence was used with the following scan parameters: TE/TR = 2.24/4.9 ms, flip angle = 8°, matrix = 256 × 58, resolution = 1.17 × 1.46 × 5 mm, bandwidth = 1150 Hz/pixel, partial Fourier in phase-encoding = 6/8, GRAPPA parallel imaging with 24 reference lines and an acceleration factor of 2.

### Statistical methods

All data are shown as mean ± SD.

### Supplementary information

Supplementary Information

Supplementary Movie 1

Supplementary Movie 2

Supplementary Movie 3

Supplementary Movie 4

Supplementary Movie 5

Supplementary Movie 6

Description of Additional Supplementary Files

## Data Availability

All data needed to evaluate the conclusions are available in the paper or the Supplementary materials. Source data for graphs are available on Figshare with the identifier 10.6084/m9.figshare.18812555.
