# Supplementary material for: MRI-compatible electromagnetic servomotor for image-guided medical robotics
Source: Commun Eng. 2022 May 26;1:4. doi: 10.1038/s44172-022-00001-y (PMC9873480; doi:10.1038/s44172-022-00001-y)
Supplement: Supplementary file 1 — Supplementary Information [file 44172_2022_1_MOESM1_ESM.pdf]

Supplementary Information for

MRI-compatible electromagnetic servomotor for image-guided medical robotics

Lorne W. Hofstetter,* Rock Hadley, Robb Merrill, Huy Pham, Gabriel C. Fine,

Dennis L. Parker

*Corresponding author. Email: lorne.hofstetter@gmail.com

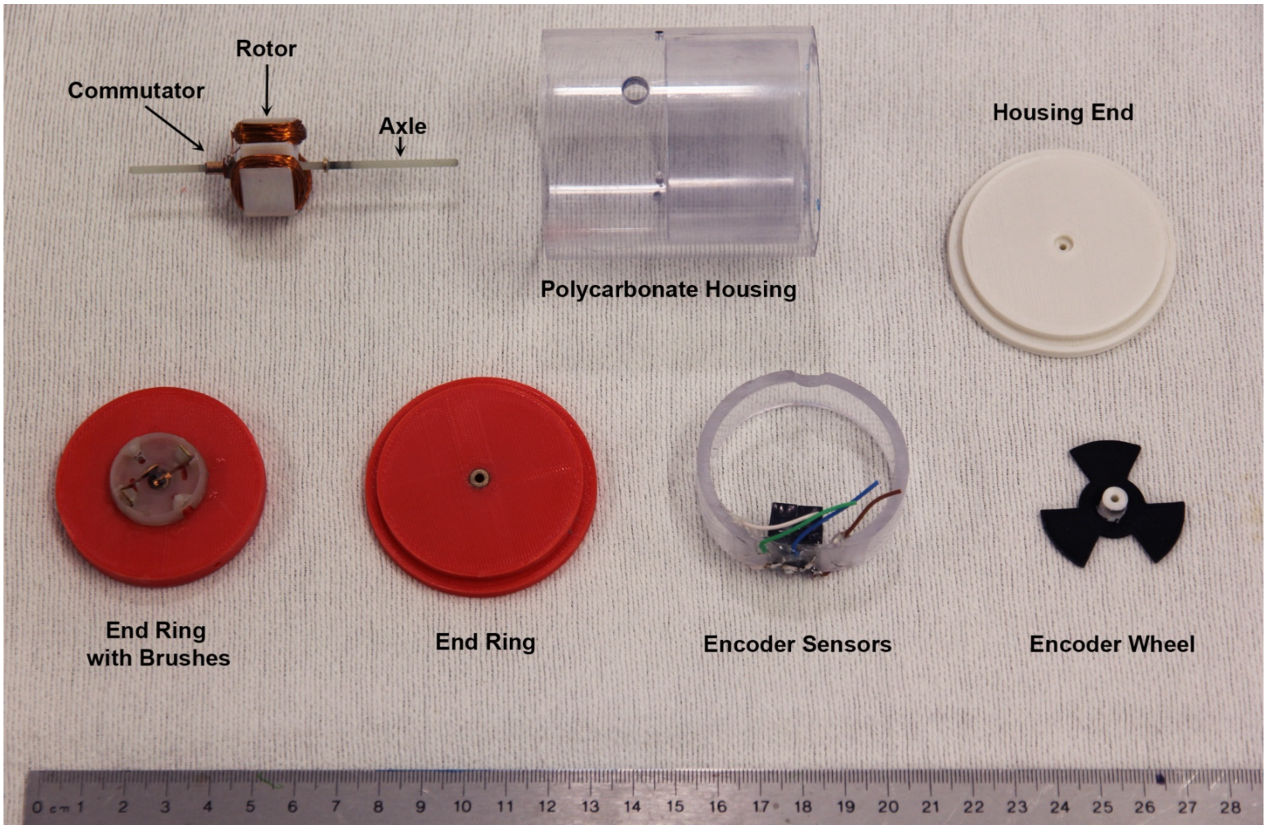

Supplementary Figure 1. Photograph of MRI-compatible DC servomotor components prior to assembly.

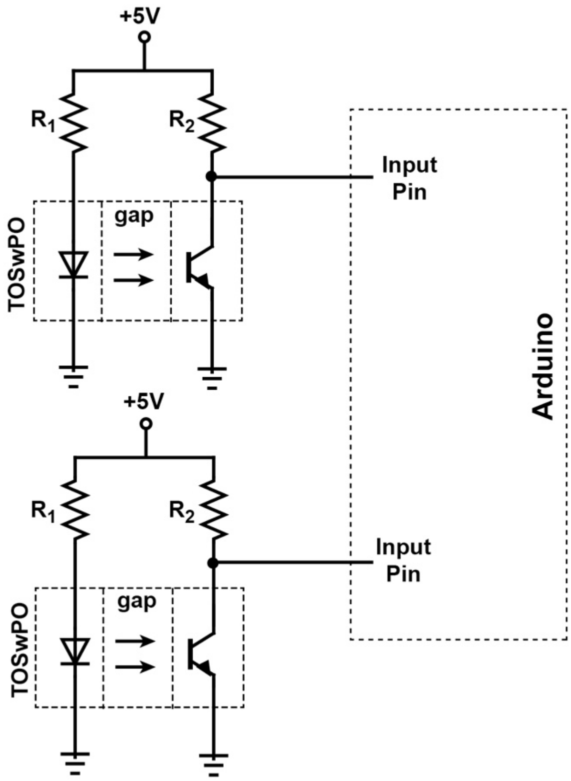

Supplementary Figure 2. Schematic of servomotor optical encoder. A Cat7 ethernet cable connects the TOSwPO sensors and resistors R_1 and R_2 where $R_1 = 180\ \Omega$ and $R_2 = 2\ k\Omega$. Encoder logic is implemented on the Arduino controller.

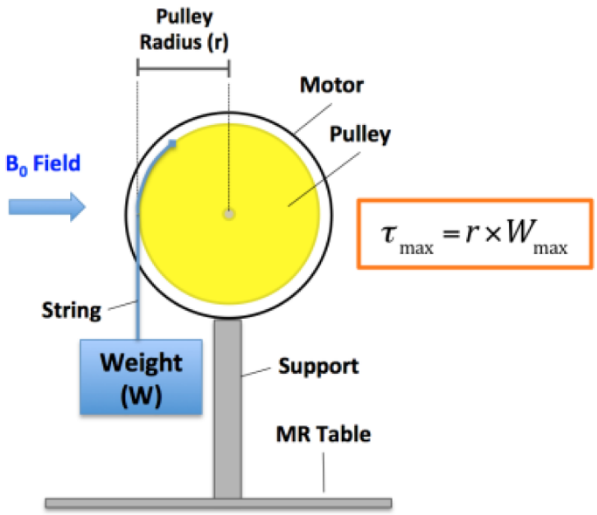

Supplementary Figure 3. Apparatus for measuring stall torque inside MRI. Stall torque (τ_{max}) was computed by determining the maximum weight (W_{max}) that could be lifted at motor stall for a given pulley radius (r).

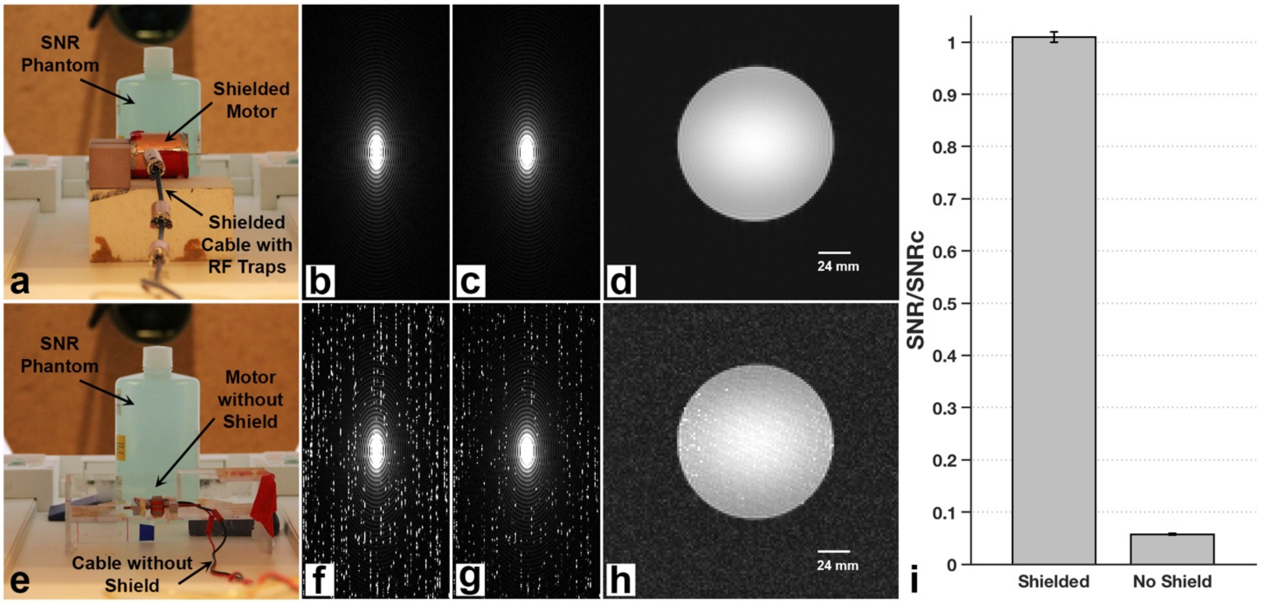

Supplementary Figure 4. Proper EMI shielding and motor design enables simultaneous operation of motor and MRI. **(a)** Photograph of the MRI-compatible servomotor located 45 cm from phantom. The raw MRI k-space data acquired during continuous servomotor operation for the two body coil receive channels is shown in **(b)** and **(c)**. The coil-combined reconstructed magnitude image is shown in **(d)**. **(e)** Photograph of an unshielded MRI-safe DC motor prototype located 45 cm from phantom. The raw MRI k-space data obtained during continuous DC motor operation for the two body coil receive channels is shown in **(f)** and **(g)**. The coil-combined reconstructed magnitude image of **(f)** and **(g)** is shown in **(h)**. Bright spike artifacts in the raw k-space data in **(f)** and **(g)** are readily apparent and image quality in **(h)** is significantly reduced when compared to **(d)**. **(i)** Impact on the measured SNR during simultaneous imaging and MRI-compatible servomotor operation was negligible. However, when the unshielded DC motor was operated simultaneous to imaging, the SNR was 5.7% of the control measurement. Data in **(i)** shows mean +/- SD.

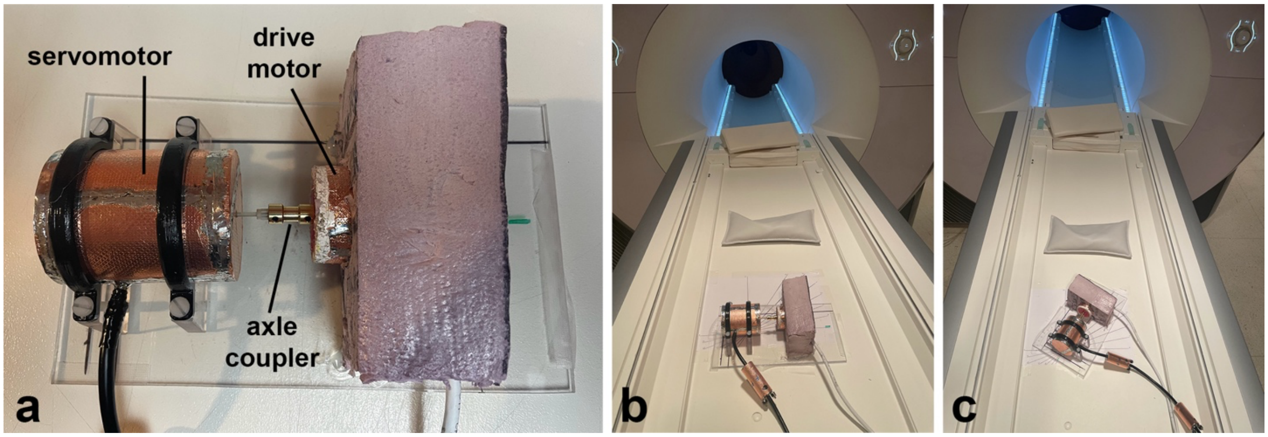

Supplementary Figure 5. Back EMF constant measurement setup. Photographs of servomotor as generator coupled to MRI-safe DC driving motor in **(a)**, servomotor axle alignment for $\theta = 90°$ in **(b)**, and $\theta = 30°$ in **(c)**.
