# Supplementary material for: MRI-compatible electromagnetic servomotor for image-guided medical robotics
Source: Commun Eng. 2022 May 26;1:4. doi: 10.1038/s44172-022-00001-y (PMC9873480; doi:10.1038/s44172-022-00001-y)
Supplement: Supplementary file 8 — Description of Additional Supplementary Files [file 44172_2022_1_MOESM8_ESM.pdf]

Description of Additional Supplementary Files

File name: Supplementary Movie 1
Description: MRI electromagnetic servomotor without EMI shielding operating in clinical 3T scanner.

File name: Supplementary Movie 2
Description: MRI electromagnetic servomotor without EMI shield operating at an angle in clinical 3T scanner.

File name: Supplementary Movie 3
Description: MRI electromagnetic servomotor maintaining desired setpoint.

File name: Supplementary Movie 4
Description: MRI-compatible biopsy introducer robot with mock introducer demonstrates simultaneous MRI and motor operation when robot oriented at an angle in MR scanner. For certain positions of the fiducial marker, an RF banding artifact due to the use of the TRUFI imaging sequence is visible.

File name: Supplementary Movie 5
Description: MRI-compatible biopsy introducer robot with mock introducer demonstrates simultaneous MRI imaging and motor operation. For certain positions of the fiducial marker, an RF banding artifact due to the use of the TRUFI imaging sequence is visible.

File name: Supplementary Movie 6
Description: Placement of 9-gauge introducer sheath using MRI-compatible biopsy introducer robot during continuous imaging.
